# An unusual presentation of neurosarcoidosis with progressive hearing loss

**DOI:** 10.11604/pamj.2017.28.213.11007

**Published:** 2017-11-07

**Authors:** Abdellah Taous, Maha Aït Berri, Mohamed Sinaa, Issam En-nafaa, Karim Nadour, Abdelhadi Rouimi

**Affiliations:** 1 Department of Neurology, Military Hospital Moulay Ismail, Meknes, Morocco; 2 Department of Anatomopathology, Military Hospital Moulay Ismail, Meknes, Morocco; 3 Department of Radiology, Military Hospital Moulay Ismail, Meknes, Morocco; 4 Department of Oto-rhino-laryngology, Military Hospital Moulay Ismail, Meknes, Morocco

**Keywords:** Hearing loss, neurosarcoidosis, rare complication

## Abstract

Sarcoidosis is a granulomatous disease of unknown etiology that can involve several organ system. Neurological manifestations are not common and mostly include cranial neuropathies. However, auricular disorders are rare and exceptionally inaugural. We describe the case of a 46-year-old lady presented with hearing loss as the initial manifestation of sarcoidosis, and aim to raise awareness of this condition, that is often associated with significant morbidity.

## Introduction

Neurosarcoidosis is a rare inflammatory disorder of the nervous system, which occurs in 7% of patients with sarcoidosis [1]. Cranial nerves are the most commonly involved, particularly the optic nerve, the trigeminal and the facial nerve [2]. However, auricular disorders are rare and exceptionally inaugural [3]. We report the case of a 46-year-old lady presented with hearing loss as the initial manifestation of sarcoidosis.

## Patient and observation

**Figure 1:**
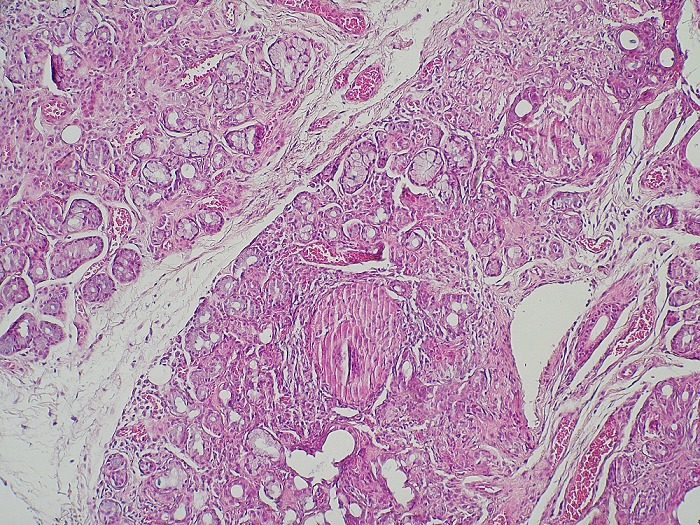
Salivary gland biopsy showing a parenchyma with non-caseating and giant-cell granulomas

A 46-year-old lady with no significant medical history, was referred to our department after a 7 months history of gradually worsening bilateral hearing loss without tinnitus, vertigo or aural discharge. Also, there were no other neurological or ocular symptoms. Clinical examination showed no neurological, otologic or ophthalmologic abnormalities. A pure tone audiogram revealed a bilateral sensorineural hearing loss, mild to moderate in the left ear and deep in the right. However, auditory brainstem response (ABR) showed normal morphology and latencies, and brain magnetic resonance imaging (MRI) with gadolinium enhancement revealed no lesions too, particularly in the cerebello-pontine angle. Routine blood tests showed a raised level of angiotensin-converting enzyme (ACE) at 253 IU (N: 12-68 IU), whereas hormonal dosages revealed no hypothalamic-pituitary axis dysfonction. There was bilateral hilar lymphadenopathy with infiltration at the thoracic computed tomography, and the salivary gland biopsy confirmed the diagnosis by finding non-caseating and giant-cell granulomas (Figure 1). The lumbar puncture showed increased proteins level (1,09g/l; upper normal limit 0,40g/l), an elevated IgG index in the cerebrospinal fluid, but no pleocytosis, According to Zajicek criteria the diagnosis of neurosarcoidosis (NS) was probable. Subsequently, the patient was given Methylprednisolone intravenously (1g/d for 3 days), before tapering oral steroids. The hearing acuity improved gradually over one year of follow up.

## Discussion

Hearing loss in systemic sarcoidosis remains a rare complication that is estimated to occur in only 1% to 7% of all patients with NS [4]. The most commonly involved cranial nerves was the optic nerve followed by the trigeminal and the facial nerve [2]. When it presents as the initial symptom of NS, whether unilateral or bilateral, it has a sudden or rapidly progressive onset 90% of the time [5]. There have been few reports describing it as one of the initial presenting symptoms of sarcoidosis in otherwise undiagnosed patients, however these were also associated with other neurological or ENT disturbances [6,7]. The diagnosis of neurosarcoidosis is based on clinical and radiological correlation in combination with a tissue biopsy [8, 9]. Although commonly requested, serum ACE has a poor predictive value in sarcoidosis. MRI is sensitive for detecting cerebral nervous system lesions of which leptomeningeal involvement with contrast enhancement is the most common finding; however, their appearances are highly variable, hence, reducing the specificity. Other findings include isolated mass lesions and diffuse inflammatory changes [8,9]. A normal MRI, as was the case of our patient does not rule out the possibility of neurosarcoidosis [8]. In large cohort of 305 patients with established neurosarcoidosis and cranial base manifestations, Carlson and al. found a meningeal enhancement only in 67% of cases [2]. Lack of abnormality on cerebral MRI despite symptoms of CNS involvement may be due to extra-cranial involvement of cranial nerves, to initially minimal perineural inflammatory infiltration or to the presence of granulomas too small to be detected by current imaging techniques.

Therefore, repetition and follow-up studies may confirme evolution of those CNS lesions [10]. The histological studies demonstrate lesions at all levels from the cochlea to the brainstem, but the main mechanism is an infiltration of the arachnoid vessels [11]. The normality of ABR in our patient suggests that the hearing impairment was of cochlear origin, whereas almost all cases of related sarcoidosis sensorineural hearing loss reported in the litterature were of retrocochlear origin likely due to a basilar meningitidis involving the cochleovestibular nerve [5]. Although evidence-based treatment guidelines derived from standard clinical trials are not available, early treatment of neurosarcoidosis is recommended. Corticosteroids are the first line treatment for neurosarcoidosis. Oral prednisone in dosages of 40-80mg/d will usually suppress disease progression and may even improve clinical manifestations. There may be some benefit of giving higher dose of corticosteroids such as Methylprednisolone 1000 mg/d intravenously for 3-5 days before the oral steroids, however the prognosis of the hearing loss is usually poor [8, 11, 12]. Our patient had the chance to get an early diagnosis and treatment, which resulted in a significant improvement in hearing.

## Conclusion

In dealing with patients with atypical or rapidly progressive hearing loss, it’s mandatory to consider to the possibility of sarcoidosis and conduct a full neurological and general examination for evidence of this condition.

## Competing interests

The authors declare no competing interests.
